# Pharmacologic Management of Pain in Children: Balancing Evidence, Safety, and Clinical Challenges

**DOI:** 10.3390/children13091181

**Published:** 2026-09-02

**Authors:** Gianmarco Marcianò, Antonio Leo, Maria Cristina Caroleo, Riccardo Torta, Vincenzo Rania, Cristina Vocca, Caterina Palleria, Lucia Muraca, Alessandro Casarella, Domenica Scumaci, Luca Gallelli

**Affiliations:** 1 Department of Health Sciences, Magna Graecia University of Catanzaro, 88100 Catanzaro, Italy; gianmarco.marciano3@gmail.com (G.M.); aleo@unicz.it (A.L.); mariacristina.caroleo@unicz.it (M.C.C.); cristina.vocca@gmail.com (C.V.); 2 CRUISE Research Center, Science of Health Department, University Magna Grecia, 88100 Catanzaro, Italy; 3 Department of Neurosciences “Rita Levi Montalcini”, University of Turin, Via Cherasco 15, 10126 Turin, Italy; riccardo.torta@unito.it; 4 Operative Unit of Clinical Pharmacology, Renato Dulbecco University Hospital, Viale Europa, 88100 Catanzaro, Italy; raniavincenzo1@gmail.com (V.R.); palleria@unicz.it (C.P.); 5 FAS@UMG Research Center, Department of Health Sciences, Magna Graecia University of Catanzaro, 88100 Catanzaro, Italy; studiomedicomuracal@gmail.com (L.M.); al.cas1993@gmail.com (A.C.); 6 Research Center on Advanced Biochemistry and Molecular Biology, Department of Experimental and Clinical Medicine, Magna Græcia University of Catanzaro, Viale Europa, 88100 Catanzaro, Italy; scumaci@unicz.it

**Keywords:** pediatric pain, analgesics, pharmacology, pharmacokinetics, neuropathic pain, nociplastic pain, children

## Abstract

**Highlights:**

**What are the main findings?**

- Pediatric pain pharmacotherapy requires a mechanism-based and developmentally informed approach.
- Evidence is strongest for acute nociceptive pain, whereas treatment of neuropathic and particularly nociplastic pain remains limited by scarce pediatric-specific trials and frequent off-label use.

**What are the implications of the main findings?**

- Treatment selection should integrate pain mechanisms, acute versus chronic presentation, age, developmental pharmacology, indication, and drug-specific safety.
- In chronic pediatric pain, functional recovery rather than complete pain elimination should represent the primary therapeutic goal, with pharmacotherapy integrated into multidisciplinary management.

**Abstract:**

Pain in children remains frequently underrecognized and undertreated despite significant advances in pediatric analgesia. The pharmacologic management of pediatric pain is challenging due to developmental differences in pharmacokinetics and pharmacodynamics, variability in drug metabolism, age-dependent responses, and the limited availability of high-quality pediatric clinical trials. This narrative review summarizes current evidence regarding the pharmacologic management of nociceptive, neuropathic, and nociplastic pain in children, integrating developmental pharmacology, efficacy data, safety considerations, and limitations of available evidence. A mechanism-based classification of pain provides a useful framework for therapeutic decision-making; however, clinical conditions often involve overlapping mechanisms requiring individualized and multidisciplinary approaches. Evidence supporting pharmacological interventions varies considerably according to pain type. Acetaminophen and NSAIDs remain the most used agents for nociceptive pain, while opioids retain a role in selected cases of moderate-to-severe pain under careful monitoring. Management of neuropathic and nociplastic pain remains particularly challenging due to limited pediatric trials, frequent off-label prescribing, and reliance on extrapolation from adult populations. Future research should focus on age-specific randomized controlled trials, pharmacokinetic and pharmacogenomic variability, long-term safety outcomes, and integration of pharmacologic treatments within multimodal pain management strategies.

## 1. Introduction

Chronic pain in children represents a significant global health issue, affecting physical, emotional, psychological, and social development. Epidemiological studies estimate that approximately 20–35% of children and adolescents experience chronic pain, with headaches, abdominal pain, and musculoskeletal disorders among the most frequently reported conditions [1,2].

Pain classification, based on underlying mechanisms, represents an important component of clinical management, allowing differentiation between nociceptive, neuropathic, and nociplastic pain. However, pediatric pain conditions frequently involve overlapping mechanisms, and mechanistic classification should be considered a framework for therapeutic reasoning rather than a strict categorization.

The most common pain mechanism in children is nociceptive pain involving tissue injury, as seen in musculoskeletal injuries, inflammatory disorders, dental conditions, and postoperative states. In children experiencing diabetic peripheral neuropathy, chemotherapy-induced peripheral neuropathy, traumatic or surgical nerve injury, or complex regional pain syndrome type II (CRPS II), a mechanistic diagnosis of neuropathic pain is postulated [3,4].

Finally, in functional abdominal pain disorders, juvenile fibromyalgia, chronic primary headache, and complex regional pain syndrome type I (CRPS I), a nociplastic pain mechanism is implicated.

Importantly, pediatric pain cannot always be attributed to a single mechanism or etiology. Nociceptive, neuropathic, and nociplastic mechanisms may coexist within the same patient and may change over the course of disease. Biological, psychological, developmental, and social factors can independently or synergistically contribute to pain persistence. Recognition of these overlapping mechanisms is particularly important in children, in whom limited verbal communication and age-dependent pain expression may further complicate phenotyping and therapeutic selection.

Despite the increasing literature on pediatric pain, a clinically relevant gap remains between available pharmacological evidence and its practical application according to pain mechanism, age, duration of pain, and indication. Pediatric practice still frequently relies on small trials, observational studies, clinical experience, or extrapolation from adult populations despite major developmental differences in pharmacokinetics, pharmacodynamics, efficacy, and safety. Therefore, this structured narrative review aims to critically evaluate current evidence on pharmacological treatment of nociceptive, neuropathic, and nociplastic pain in children, distinguishing acute from chronic pain whenever supported by available evidence and integrating age-related pharmacology, clinical indications, efficacy, safety, regulatory limitations, and major evidence gaps.

## 2. Methods

### Literature Search Strategy

This study was designed as a structured narrative review. A literature search was conducted in PubMed/MEDLINE and the Cochrane Library for publications available up to 2026. The search combined terms related to the population (“child”, “children”, “adolescent”, “pediatric”), pain mechanisms (“nociceptive pain”, “neuropathic pain”, “nociplastic pain”, “chronic pain”, “acute pain”), and pharmacological interventions (“analgesics”, “acetaminophen”, “paracetamol”, “NSAIDs”, “opioids”, “gabapentin”, “pregabalin”, “antidepressants”, “amitriptyline”, “duloxetine”, “melatonin”), using Boolean operators as appropriate. Randomized controlled trials, observational studies, systematic reviews, meta-analyses, regulatory documents, and major clinical guidelines published in English were considered. Priority was given to pediatric-specific evidence; adult evidence was considered only when pediatric data were unavailable and when relevant to explain current off-label practice, developmental pharmacology, or safety. Disease-specific protocols for acute migraine and localized organic gastrointestinal conditions were excluded to maintain focus on core systemic analgesic strategies. Drugs were selected according to their reported use in pediatric pain management, availability of pediatric clinical evidence, inclusion in relevant recommendations, and pharmacological relevance to nociceptive, neuropathic, or nociplastic pain. Reference lists of relevant reviews and guidelines were additionally screened. Because the objective was a clinically oriented synthesis rather than a systematic estimate of treatment effect, the review was not designed as a PRISMA systematic review.

## 3. Pediatric Pharmacology: Pharmacokinetics and Pharmacodynamics

### 3.1. Pharmacokinetics

Pediatric pharmacology is a rapidly evolving field where drug responses are shaped by dynamic developmental changes occurring from birth through adolescence. Pediatric patients cannot be considered merely as “small adults”; their absorption, distribution, metabolism, elimination (ADME), and pharmacodynamic profiles undergo progressive maturation (Table 1). This results in highly age-dependent variations in drug exposure and clinical efficacy [5,6].

These developmental trajectories are critically relevant in pain management, where imprecise dosing can lead to either subtherapeutic analgesia or an increased risk of severe adverse effects. Consequently, pharmacological decisions in pediatric pain management must carefully integrate age, body weight, developmental stage, organ maturation, and the specific pharmacokinetic properties of each analgesic agent.

#### 3.1.1. Absorption

Drug absorption is significantly modulated by developmental changes in gastrointestinal physiology. Neonates and infants exhibit a higher gastric pH compared to older children and adults. This alteration modifies the bioavailability of orally administered drugs, enhancing the absorption of acid-labile compounds while potentially reducing that of weak acids. Furthermore, gastric emptying and intestinal motility are prolonged or erratic during early infancy, which can delay the onset of action of oral analgesics—a critical factor when rapid pain relief is required. Beyond the gastrointestinal tract, transdermal absorption varies substantially across pediatric age groups; the thinner *stratum corneum* and increased skin hydration characteristic of neonates and infants can markedly enhance systemic exposure to topical agents (e.g., lidocaine), necessitating rigorous dosing evaluation to prevent systemic toxicity [7].

#### 3.1.2. Distribution

Age-dependent shifts in body composition directly influence drug distribution. Neonates possess a remarkably higher total body water content, reaching approximately 80% of total body weight. This results in a larger volume of distribution ($V_{d}$) for hydrophilic drugs, such as morphine, which may necessitate higher initial weight-based doses to achieve therapeutic plasma concentrations.

Conversely, lower plasma protein concentrations (specifically albumin and $\alpha_{1}$-acid glycoprotein) in younger cohorts increase the free (unbound) fraction of highly protein-bound medications. This pharmacologically active fraction can enhance both therapeutic efficacy and the risk of acute toxicity, reinforcing the absolute need for age-adjusted dosing strategies [7].

#### 3.1.3. Metabolism

Hepatic drug metabolism undergoes profound ontogeny during childhood. The activity of the Cytochrome P450 (CYP450) enzyme system changes progressively from birth through adolescence. While CYP3A7 activity is predominant during fetal life and early infancy, it rapidly declines postpartum as CYP3A4, CYP2D6, and CYP2C9 activities gradually mature.

These developmental patterns are highly relevant for analgesics relying on hepatic clearance. For instance, the ontogeny and genetic polymorphism of CYP2D6 drastically influence individual exposure and clinical response to prodrug opioids like codeine and tramadol, as well as oxycodone, leading to unpredictable efficacy or severe adverse events (e.g., respiratory depression).

Phase II conjugation pathways, particularly glucuronidation, exhibit similar maturation profiles: UDP-glucuronosyltransferases (UGTs) are immature at birth and reach adult-like capacity progressively during childhood. This directly impacts the clearance and metabolite profiles of cornerstone pediatric analgesics, including paracetamol (acetaminophen) and morphine [7,8].

#### 3.1.4. Elimination

Renal elimination is a primary determinant of pediatric drug exposure and clearance. The glomerular filtration rate (GFR) is restricted to approximately 30–40% of adult values at birth, increases sharply during the first few weeks of life, and typically reaches adult levels within the first year. Tubular secretion and reabsorption mature at an even slower rate.

Consequently, analgesics and co-analgesics primarily cleared via renal excretion require precise, age-stratified dosing intervals to avoid drug accumulation. This is particularly critical for medications such as gabapentin, pregabalin, and active opioid metabolites (e.g., morphine-6-glucuronide), where impaired clearance directly correlates with an increased risk of neurotoxicity or respiratory depression [9].

### 3.2. Pharmacodynamics

While developmental pharmacokinetic differences are increasingly well-characterized, pediatric pharmacodynamics—the relationship between drug concentration and clinical effect, encompassing both therapeutic efficacy and toxicity—remains less extensively understood.

In pediatric patients, pharmacodynamic responses can deviate significantly from adult models due to age-dependent variations in receptor expression, density, and binding affinity, as well as immature intracellular signaling cascades and evolving ion channel distribution [10]. Furthermore, the progressive maturation and myelination of neural circuits involved in nociception and endogenous pain modulation profoundly alter how a child perceives both pain and analgesic interventions.

These neurodevelopmental discrepancies underscore why the direct extrapolation of adult analgesic efficacy and safety data to pediatric cohorts can be profoundly misleading. An analgesic that is highly effective and well-tolerated in adults may exhibit blunted efficacy, altered potency, or unexpected toxicity in children due to these ontogenetic variations in target organ sensitivity and central nervous system (CNS) maturation.

Ultimately, safe and effective pediatric pain pharmacotherapy demands a comprehensive integration of both pharmacokinetic and pharmacodynamic principles, rather than a simplistic scaling of adult doses based solely on body weight or surface area. Adopting a strict developmental pharmacology framework is imperative to optimize analgesia while mitigating the risk of adverse drug reactions.

## 4. Nociceptive Pain Phenotype

Nociceptive pain is the most prevalent pain phenotype in the pediatric population, arising from the activation of peripheral nociceptors in response to actual or threatened tissue injury and subsequent inflammation. In children, it is characteristically driven by musculoskeletal trauma, acute inflammatory conditions, dental disease, and postoperative states [11].

Although typically considered more straightforward to manage than neuropathic or nociplastic pain, pediatric nociceptive pain still presents substantial clinical challenges. These challenges include highly variable age-dependent pain expressions, the inherent difficulties of objective pain assessment in non-verbal cohorts, and a historical paucity of high-quality, pediatric-specific pharmacological trials in acute trauma and surgical settings.

Among chronic and recurrent manifestations, pediatric rheumatologic conditions—most notably juvenile idiopathic arthritis (JIA)—represent a critical etiology of persistent nociceptive pain [12]. If inadequately managed, this ongoing inflammatory nociception can severely impair physical development, joint integrity, and long-term functional outcomes. In the acute setting, dental pain and postoperative pain remain the most ubiquitous manifestations of nociceptive pain encountered in routine pediatric practice, both requiring prompt multimodal intervention to prevent central sensitization [13,14].

## 5. Pharmacological Management of Nociceptive Pain: Non-Opioid Analgesics

### 5.1. Acetaminophen

Acetaminophen remains the most ubiquitously prescribed analgesic and antipyretic agent in pediatric medicine, serving as a first-line intervention for mild-to-moderate nociceptive pain. While its precise mechanism of action remains partially elusive, it is understood to involve the central inhibition of prostaglandin synthesis alongside the modulation of endogenous serotonergic and endocannabinoid pathways [15].

Despite its pervasive clinical use, the quality of evidence demonstrating the analgesic superiority of acetaminophen across various pediatric conditions is surprisingly constrained. Most randomized controlled trials (RCTs) indicate comparable or only modest differences in pain scores when acetaminophen is compared to non-steroidal anti-inflammatory drugs (NSAIDs), particularly in acute inflammatory states. For instance, a systematic review focusing on pediatric acute otitis media reported low-certainty evidence regarding its efficacy, failing to demonstrate a clear therapeutic superiority over ibuprofen, whether administered as monotherapy or in combination [16]. This highlights a critical clinical paradigm: acetaminophen is predominantly selected as a first-line therapy due to its favorable and well-characterized safety profile rather than robust evidence of superior analgesic potency.

In perioperative care, acetaminophen effectively reduces postoperative pain intensity and exerts a documented opioid-sparing effect. However, its dose–response relationship requires careful navigation; higher doses do not linearly translate into enhanced analgesia but significantly escalate the risk of adverse effects, such as postoperative nausea and vomiting (PONV) [17].

Regarding the route of administration, meta-analytic data show no clinically meaningful differences in analgesic efficacy among oral, intravenous (IV), and rectal routes in most pediatric postoperative settings [18,19]. While rectal administration is widely used, rectal absorption in children is characteristically slow, erratic, and variable; thus, oral or IV routes are preferred whenever accessible to ensure predictable therapeutic levels and avoid accidental cumulative dosing [20].

From a safety perspective, acetaminophen is generally well tolerated when administered within recommended therapeutic ranges. Pediatric dosing is typically 10–15 mg/kg per dose every 4–6 h. The maximum daily exposure should be defined according to age, clinical setting, duration of treatment, and applicable national recommendations rather than presented as a single universal value. Repeated supratherapeutic administration, accidental overdose, and duplication across combination products represent important causes of preventable hepatotoxicity [21].

### 5.2. NSAIDs

NSAIDs constitute the cornerstone of multimodal management for pediatric nociceptive pain with a prominent inflammatory component. Their primary mechanism revolves around the inhibition of cyclooxygenase enzymes (COX-1 and/or COX-2), thereby halting the cascade of prostaglandin synthesis responsible for peripheral nociceptor sensitization, inflammation, and pyrexia [4,22].

Compared to acetaminophen, NSAIDs generally offer superior analgesic efficacy in inflammatory states owing to these peripheral anti-inflammatory properties. Nevertheless, their implementation in pediatric cohorts demands a meticulous evaluation of age restrictions, available formulations, and drug-specific safety profiles. NSAIDs collectively represent first-line therapy for inflammatory nociceptive pain in children when renal function, hydration status, and gastrointestinal risk factors are appropriate (Table 2). However, their use should always be individualized based on age, clinical condition, duration of therapy, and comorbidities.

#### 5.2.1. Ibuprofen

Ibuprofen is the most extensively investigated NSAID in pediatrics and is regulatory-approved for early infancy (typically from 3 months of age, depending on regional jurisdictions). Systematic reviews and RCTs confirm that ibuprofen provides highly effective short-term analgesia for acute pediatric pain, especially musculoskeletal injuries [23].

Crucially, ibuprofen exhibits an excellent safety profile when properly titrated. Concerns regarding gastrointestinal hemorrhage or clinically significant platelet dysfunction remain largely theoretical in otherwise healthy pediatric populations, though a cautious approach is warranted in perioperative environments or in children with pre-existing risk factors (e.g., dehydration, coagulopathies).

#### 5.2.2. Naproxen

Naproxen has established clinical efficacy in the management of Juvenile Idiopathic Arthritis (JIA), where it significantly mitigates pain, restores joint function, and reduces global disease activity compared to placebo [24,25,26]. Pharmacokinetically, naproxen features a prolonged half-life relative to ibuprofen, enabling a less frequent dosing regimen (twice daily) that significantly enhances treatment adherence in chronic conditions. Regulatory approval varies globally; in Europe, it is primarily reserved for adolescents, with pediatric liquid formulations limited to specific, severe rheumatologic indications in younger children. Consequently, naproxen is positioned almost exclusively for chronic inflammatory conditions rather than acute pain management.

#### 5.2.3. Ketorolac

Ketorolac is a highly potent NSAID characterized by robust analgesic efficacy and substantial opioid-sparing properties, making it highly valuable in acute postoperative pathways. In pediatrics, its utilization is tightly restricted to short-term, hospital-based intravenous or oral administration due to class-related risks of gastrointestinal ulceration, acute kidney injury (AKI), and surgical site bleeding [27,28]. Ultimately, ketorolac must be categorized as a second-line, inpatient-only NSAID rather than a routine outpatient analgesic.

#### 5.2.4. Diclofenac

The evidence base supporting diclofenac in pediatric pain management is substantially weaker than that of ibuprofen. Available pediatric literature is compromised by small sample sizes, clinical heterogeneity, and a moderate-to-high risk of bias [29,30].

Diclofenac should be approached with caution and reserved for selected clinical scenarios where first-line NSAIDs are contraindicated or unavailable.

#### 5.2.5. Ketoprofen Lysine Salt

Ketoprofen lysine salt has demonstrated promising efficacy in managing pediatric postoperative pain, with several trials suggesting superior analgesic outcomes compared to acetaminophen [31]. The lysine salt formulation accelerates absorption, leading to a faster onset of action. It remains a viable option for short-term acute pain control, provided it is balanced against standard NSAID class contraindications.

#### 5.2.6. Meloxicam

Meloxicam has been evaluated primarily in juvenile idiopathic arthritis and other chronic inflammatory pediatric conditions. Its longer elimination half-life may permit once-daily administration, but age authorization and formulation availability vary by jurisdiction. It should therefore be considered an indication-specific alternative rather than a routine first-line drug for acute pediatric pain [24].

### 5.3. Opioids

A definitive distinction must be maintained between acute pain management and long-term chronic pain strategies in pediatric patients. Except for oncological pain and pediatric palliative care, the use of opioids is strictly avoided in the management of pediatric chronic non-cancer pain (CNCP) (e.g., fibromyalgia, chronic musculoskeletal pain, and functional abdominal syndromes).

The rationale for this restriction is twofold:

1. Lack of Long-Term Efficacy: Current clinical evidence fails to demonstrate any long-term functional or analgesic benefit of opioid therapy for chronic non-cancer pain in children.
2. Severe Risks: Prolonged exposure in developing organisms significantly escalates the risk of opioid-induced hyperalgesia (OIH)—where the drug paradoxically increases pain sensitivity—alongside rapid tolerance, physical dependence, and psychological addiction (opioid use disorder).

Consequently, modern pediatric guidelines mandate that chronic non-cancer pain must be addressed through a rehabilitative, multidisciplinary, and biopsychosocial approach, prioritizing non-pharmacological interventions (such as cognitive–behavioral therapy and physical rehabilitation) combined, when necessary, with non-opioid co-analgesics.

Furthermore, contemporary pediatric guidelines heavily emphasize the risk of accidental ingestion and the potential for prescription opioid misuse during adolescence. Cautious prescribing practices—characterized by the lowest effective dose, restricted prescription quantities for outpatient discharge, and structured transition plans back to non-opioid baselines—are imperative to optimize pediatric patient safety.

Caution is required for codeine and tramadol because their analgesic activity depends substantially on CYP2D6-mediated metabolism, resulting in marked interindividual variability in exposure to active opioid metabolites. Regulatory authorities have introduced age- and procedure-specific restrictions because ultra-rapid metabolizers may develop potentially life-threatening opioid toxicity and respiratory depression. Codeine is contraindicated in children younger than 12 years and should not be used after tonsillectomy/adenoidectomy in patients younger than 18 years; tramadol is likewise contraindicated in children younger than 12 years and after tonsillectomy/adenoidectomy in patients younger than 18 years under current US regulatory restrictions. European restrictions are also age- and indication-specific. These agents should therefore not be considered interchangeable with morphine or other directly active opioids, and any use must comply with current regional labeling and regulatory guidance.

In palliative and acute moderate-to-severe pain, commonly used opioids include morphine, oxycodone, fentanyl, and hydromorphone (Table 3):

- Morphine remains the reference standard for comparative evaluation [32]. Clinical studies demonstrate effective analgesia but also highlight clinically relevant risks, particularly respiratory depression in vulnerable populations such as children with sleep-disordered breathing.
- Oxycodone, evaluated as an alternative to morphine, has shown comparable analgesic efficacy in pediatric surgical populations [33,34]. However, interindividual variability in response due to CYP2D6-mediated metabolism remains a significant limitation.
- Fentanyl shows comparable efficacy to other strong opioids, with practical advantages in rapid titration and transdermal or transmucosal delivery in chronic/palliative care [35,36,37,38].
- Hydromorphone has been studied as an alternative opioid, but available evidence does not consistently demonstrate superior safety or efficacy compared with morphine [39,40].

## 6. Neuropathic Pain Phenotype

Neuropathic pain in the pediatric population is a complex, distressing, and often debilitating condition arising as a direct consequence of a lesion or disease affecting the somatosensory nervous system. The clinical presentation is highly heterogeneous and characteristically encompasses burning pain, electric shock-like sensations, paroxysmal shooting pain, mechanical or thermal allodynia, hyperalgesia, and paradoxical sensory deficits. However, the objective characterization and verbal description of these symptoms in pediatric cohorts are frequently hindered by cognitive development and communication barriers, particularly in pre-verbal or early childhood stages. Nonverbal signs of pain, like facial expressions and reactions to stimuli, may be useful for a clearer diagnosis. This inherent diagnostic challenge significantly contributes to widespread underdiagnosis, delayed intervention, or clinical misclassification [3].

The etiological spectrum includes diabetic peripheral neuropathy, chemotherapy-induced peripheral neuropathy (CIPN), post-infectious neuralgias, traumatic or iatrogenic nerve injury, and complex regional pain syndrome type II [41,42,43,44]. In children and adolescents, chronic neuropathic pain is tightly coupled with profound functional impairment, severe sleep fragmentation, and psychological distress (such as anxiety and depression), which synergistically amplify central pain perception through a vicious cycle. Crucially, pediatric neuropathic pain is rarely a purely isolated biological phenomenon; it is sustained by an intricate interplay between peripheral nerve injury, spinal/cortical central sensitization, and prominent psychosocial factors. Consequently, pharmacological interventions must never be deployed as standalone therapies but rather integrated into a comprehensive, multidisciplinary, and multimodal biopsychosocial rehabilitation framework.

### 6.1. Neuropathic Co-Analgesics: Gabapentinoids and Antidepressants

#### 6.1.1. Gabapentinoids

Gabapentinoids, principally gabapentin and pregabalin, are extensively utilized in pediatric clinical practice for neuropathic pain modulation; however, the quality of high-level supporting evidence remains restricted (Table 4).

Mechanistically, these structural analogs of gamma-aminobutyric acid (GABA) do not bind to GABA receptors; instead, they selectively bind to the α2δ subunit of voltage-gated calcium channels (VGCCs) within the central nervous system. By downregulating these channels, they decrease calcium influx into presynaptic terminals, thereby inhibiting the exocytosis of excitatory neurotransmitters (such as glutamate and substance P) [4].

From a pharmacokinetic perspective, the two agents differ significantly:

- Gabapentin exhibits non-linear, saturable zero-order absorption via the L-amino acid transport system, necessitating strict multi-daily dosing regimens (q8h) and complex titration schedules to achieve therapeutic steady-state concentrations.
- Pregabalin displays a highly predictable, linear pharmacokinetic profile with rapid absorption and superior bioavailability, allowing for simplified dosing schedules.
- Both medications are exclusively eliminated via renal excretion without undergoing hepatic metabolism, rendering them highly dependent on renal function and necessitating precise dose adjustments in patients with renal impairment. Somnolence, dizziness, peripheral edema, and subtle cognitive/behavioral changes represent the most ubiquitous treatment-emergent adverse events reported in pediatric cohorts [45]. On the other hand, such molecules demonstrate an advantageous action in reducing anxiety and improving sleep, factors also involved in pain control [46].

Clinical trials evaluating gabapentinoids in children yield highly heterogeneous and conflicting outcomes. While some randomized trials and open-label extensions suggest a tangible therapeutic benefit in localized neuropathic conditions, including CRPS and post-amputation phantom limb pain, other robust trials—particularly those evaluating CIPN—failed to demonstrate any statistically significant superiority over placebo or conventional multimodal regimens [47]. This marked inconsistency suggests that while gabapentinoids may offer targeted efficacy in highly specific pediatric neuropathic sub-phenotypes, current high-quality evidence does not justify their blanket endorsement as universal first-line agents.

Pregabalin has been evaluated in smaller, specialized pediatric studies, primarily involving oncological populations suffering from refractory neuropathic pain, with investigators reporting modest improvements in pain scores and acceptable short-term tolerability [48,49]. However, these findings are intrinsically limited by their open-label designs, small cohort sizes, and lack of active comparators, which severely restricts their generalizability. Ultimately, recent systematic reviews and meta-analyses underscore a profound paucity of high-certainty, high-quality evidence validating the efficacy of gabapentinoids for chronic pediatric neuropathic pain [50].

#### 6.1.2. Antidepressants

Antidepressants offer a valuable option in pain management, as they act on mood, anxiety, pain, and stress. Furthermore, they control the hypothalamic–pituitary–adrenal (HPA) axis, modulating stress hormones, vasopressin, cytokines, and, in part, the autonomic nervous system (ANS) [51].

#### 6.1.3. Tricyclic Antidepressants (TCAs)

Tricyclic antidepressants, with amitriptyline serving as the primary representative, are frequently prescribed for pediatric neuropathic pain, a practice largely driven by the historical extrapolation of adult data. Their multifaceted mechanism of action includes the inhibition of presynaptic serotonin (5-HT) and norepinephrine (NE) reuptake pumps, alongside the blockade of voltage-gated sodium channels. This synergistic pharmacology serves to reinforce and restore the impaired descending inhibitory pain pathways within the spinal cord [52].

Despite their ubiquity in routine pediatric management, high-quality pediatric RCTs evaluating TCAs remain sparse [53].

Furthermore, the clinical utility of TCAs is constrained by their adverse effect profile. Anticholinergic symptoms (dry mouth, urinary retention, blurred vision, constipation), profound sedation, and the risk of cardiac conduction abnormalities—most notably dose-dependent prolongation of the corrected QT interval (QTc)—mandate baseline electrocardiograms (ECGs) and careful dose titration. The use at low doses may not favor an antidepressant effect but rather antalgic and sedative activity [54,55].

#### 6.1.4. Serotonin-Norepinephrine Reuptake Inhibitors (SNRIs)

SNRIs, most notably duloxetine, possess a more favorable safety profile and stronger evidence-based support in adult chronic pain guidelines compared to TCAs; however, they remain insufficiently investigated in pediatrics [56,57]. In preliminary reports, duloxetine has been associated with dual benefits, mitigating both neuropathic pain scores and comorbid mood disturbances (such as anxiety and depressive symptoms) in adolescents. The action on pain, compared to selective serotonin reuptake inhibitors (SSRIs), is mainly determined by the inhibition of norepinephrine reuptake, which suppresses descending pain pathways [58]. Nonetheless, gastrointestinal adverse events (nausea, abdominal pain), insomnia, and acute withdrawal syndromes upon discontinuation frequently lead to premature treatment termination.

#### 6.1.5. Topical and Other Pharmacologic Options

Topical therapies represent an attractive option for localized neuropathic pain due to their favorable systemic safety profile.

Lidocaine 5% patches have shown moderate efficacy in pediatric neuropathic pain conditions, including sickle cell disease–associated pain and localized neuropathic syndromes [59,60].

Capsaicin, a TRPV1 receptor agonist, has demonstrated efficacy in adult neuropathic pain conditions, but pediatric evidence remains lacking, and its use in children remains experimental [4].

## 7. Pharmacologic Treatment of Nociplastic Pain

Pharmacological management of nociplastic pain in children and adolescents presents a profound clinical challenge. Under nociplastic conditions—where pain arises from altered nociceptive processing and central sensitization without objective evidence of actual or threatened tissue damage or somatosensory lesions—pharmacotherapy must be conceptualized as an adjunct rather than a primary treatment modality [61,62,63]. The level of evidence supporting these interventions in pediatric nociplastic pain is generally low-to-moderate, and clinical responses remain highly heterogeneous. Consequently, rather than targeting a structural lesion, the pharmacological rationale relies on agents that modulate dysregulated central neurotransmission [64,65,66,67].

Commonly used drug classes include TCAs, SNRIs, gabapentinoids, and agents targeting sleep regulation such as melatonin [68].

### 7.1. Amitriptyline

Frequently used in pediatric functional pain syndromes and chronic headache disorders. Available data suggest modest benefit in some patients, particularly in improving sleep quality and functional outcomes rather than producing consistent reductions in pain intensity [69]. This dissociation between pain reduction and functional improvement is a key consideration in pediatric nociplastic pain management.

### 7.2. Duloxetine

In adolescents with juvenile fibromyalgia, randomized trials have shown no statistically significant superiority over placebo for primary pain outcomes, although several secondary functional measures showed modest improvement [56,70]. This suggests that duloxetine may have a role in selected patients, particularly when pain coexists with mood disorders or significant functional impairment.

### 7.3. Gabapentin and Pregabalin

Sometimes used in nociplastic pain syndromes when there is overlap with neuropathic-like symptoms. However, evidence supporting their efficacy in pure nociplastic pain is limited, and clinical trials in pediatric fibromyalgia have generally failed to demonstrate robust benefits over placebo [71].

### 7.4. Melatonin

Melatonin has emerged as a valuable, low-risk adjunctive agent in pediatric nociplastic pain management, particularly for patients presenting with functional abdominal pain disorders heavily compounded by sleep disturbances. Clinical data suggest that melatonin (ranging from specialized neuroprotective protocols in neonatal intensive care to standard weight-adjusted doses of 3–5 mg in patients aged 4–18 years) offers tangible benefits in optimizing sleep efficiency and mitigating pain-related psychological distress [72,73].

Given its exceptionally favorable safety profile, melatonin represents a viable component of a comprehensive therapeutic strategy aimed at restoring circadian rhythms and breaking the pain–insomnia cycle.

## 8. Summary of Pharmacological Goals

Pharmacological interventions in pediatric nociplastic pain should be strictly viewed as supportive rather than curative. Medications should never be prescribed with the expectation of complete pain elimination. Instead, pharmacotherapy should be deliberately targeted to achieve specific, objective biopsychosocial goals:

- Central Modulation: Attenuating hyperexcitable central pain-processing circuits.
- Sleep Optimization: Restoring restorative sleep stages to break the pain–insomnia cycle.
- Comorbid Mitigation: Reducing the burden of co-existing anxiety, hypervigilance, or depressive symptoms.
- Rehabilitative Facilitation: Lowering the chemical barrier of pain to allow active engagement in physical and rehabilitation strategies.

## 9. The Cornerstone: Multidisciplinary, Function-Oriented Management

The definitive gold standard for pediatric nociplastic pain management is a coordinated, multidisciplinary, and strictly function-oriented approach. Non-pharmacological strategies constitute the absolute cornerstone of treatment, while drugs serve merely as facilitators. This integrated framework encompasses:

- Cognitive-Behavioral Therapy (CBT): Targeting maladaptive pain coping mechanisms, catastrophic thinking, and central hypervigilance.
- Graded Physical Activity and Physiotherapy: Overcoming kinesiophobia (fear of movement), reversing neuromuscular deconditioning, and utilizing descending inhibitory pathways through exercise.
- Sleep Hygiene Optimization: Implementing non-pharmacological behavioral modifications to stabilize circadian rhythms.
- Family-Centered Interventions: Educating parents to shift focus away from pain behaviors and toward the reinforcement of functional gains.
- Structured School Reintegration: Preventing social isolation and academic decay by establishing structured school attendance plans.

From a clinical perspective, this framework suggests a sequential approach: (1) identify the predominant pain mechanism while recognizing possible overlap; (2) distinguish acute from chronic pain; (3) define age, developmental stage, comorbidities, and functional impairment; (4) select pharmacological treatment according to mechanism-specific evidence and pediatric safety; and (5) reassess treatment according to functional outcomes rather than pain intensity alone in chronic pain (Table 5). This framework should not be interpreted as a formal clinical guideline but may provide a basis for future pediatric consensus statements and prospective validation studies.

## 10. Discussion

This review highlights a critical gradient of evidence strength in pediatric pain pharmacotherapy across distinct clinical phenotypes [7,74]: higher certainty in acute nociceptive pain [75,76], moderate-to-low in neuropathic pain [57], and very low in nociplastic pain [63,77]. Recognizing this gradient is vital for guiding realistic clinical expectations, regulatory decisions, and off-label prescribing practices in pediatric care [11,78].

The primary barrier to high-certainty evidence in pediatric chronic pain lies in clinical trial feasibility. While acute nociceptive pain trials benefit from short observation windows and standardized surgical models, chronic neuropathic and nociplastic conditions involve complex neurodevelopmental, psychological, and environmental interactions that resist simple trial designs. Consequently, clinical management in children relies heavily on extrapolation from adult literature, exposing pediatric populations to off-label drug exposure without robust pediatric PK/PD validation [57,74,75].

A pivotal takeaway for clinical practice is the necessity of redefining treatment endpoints in chronic and nociplastic pain. While complete pain eradication remains a primary goal in acute nociceptive pain, success in chronic pediatric pain must be measured through functional restoration—such as school attendance, sleep quality normalization, reduced anxiety, and improved quality of life. Pharmacotherapy should be conceptualized not as a solitary cure but as a facilitator to enable active participation in physical rehabilitation and psychological therapies [3,61,67].

Addressing existing evidence gaps requires methodological innovation. Future research priorities must include age-stratified randomized controlled trials, long-term safety registries for centrally acting drugs (gabapentinoids and SNRIs), integration of pharmacogenomic profiling to mitigate CYP2D6/CYP2C9 metabolic variability, and standardized functional outcome measures across pediatric trial networks [50,53,56].

Compared with reviews centered on individual analgesics or single pediatric pain conditions, the present review integrates developmental pharmacology with a mechanism-based classification and explicitly considers the different therapeutic goals of acute and chronic pain. This approach highlights a clinically relevant evidence gradient: pharmacological choices are relatively well supported in acute nociceptive pain, whereas neuropathic and especially nociplastic pain remain characterized by heterogeneous studies, off-label prescribing, and limited long-term pediatric safety data.

## 11. Strengths and Limitations

A major strength of this review is the integration of developmental pharmacology with a mechanism-based classification of pediatric pain, allowing pharmacological evidence to be interpreted according to nociceptive, neuropathic, and nociplastic phenotypes rather than drug class alone. Nevertheless, several limitations should be acknowledged. First, this is a structured narrative rather than a systematic review, and study selection may therefore be subject to selection bias. Second, pediatric evidence remains heterogeneous and limited for several pharmacological classes, particularly for chronic neuropathic and nociplastic pain. Third, age-specific dosing and regulatory authorization vary among countries, limiting the universal applicability of individual recommendations. Finally, for several off-label treatments, current pediatric practice remains partly based on extrapolation from adult studies, and long-term safety data are limited.

## 12. Conclusions

The management of pain in pediatric populations cannot be reduced to a mere pharmacological prescription, even when tailored to age and weight. It must encompass the emotional, developmental, and relational factors that modulate pain perception and therapeutic response. In conclusion, pediatric pain management remains an area where clinical demands routinely outpace the empirical evidence available to guide them safely. Mitigating this vulnerability requires a coordinated conceptual shift toward individualized, developmentally informed, and function-oriented multidisciplinary strategies.

## Figures and Tables

**Table 1:** Key developmental pharmacokinetic (ADME) parameters in pediatrics.

Parameter	Neonates & Infants	Children & Adolescents	Clinical Impact on Analgesia
Absorption	Elevated gastric pH; delayed gastric emptying; increased skin permeability.	Gastrointestinal pH and motility approach adult values.	Delayed onset of oral analgesics; heightened systemic absorption risk for topical agents (e.g., lidocaine).
Distribution	Higher total body water (~80%); reduced plasma protein binding (albumin/AAG).	Body water content decreases; protein binding reaches adult levels.	Increased Vd for hydrophilic drugs (e.g., morphine); higher free active fraction of protein-bound drugs.
Metabolism	Immature CYP450 (CYP2D6, CYP3A4) and Phase II UGT pathways.	Rapid enzyme maturation; clearance per kg may exceed adult levels.	Unpredictable prodrug activation (e.g., codeine, tramadol); altered morphine/paracetamol clearance.
Elimination	Reduced GFR (30–40% of adult capacity); immature tubular function.	GFR reaches adult values by 1 year of age.	Risk of accumulation for renally cleared drugs (gabapentin, active opioid metabolites).

**Table 2:** Dosing, indications, and half-lives of common pediatric NSAIDs.

Agent	Recommended Pediatric Dose	Plasma Half-Life	Primary Indications & Regulatory Considerations	Age/Regulatory Notes
Ibuprofen	5–10 mg/kg q6–8 h (max 40 mg/kg/day)	1.8–2.0 h	First-line for acute nociceptive/inflammatory pain; approved from 3 months of age.	From ~3 months where locally authorized; avoid in significant dehydration/renal risk.
Naproxen	5–10 mg/kg q12 h (max 1000 mg/day)	12–15 h	Juvenile Idiopathic Arthritis (JIA) and chronic inflammatory pain; twice-daily dosing.	Mainly older children/adolescents; pediatric authorization varies by jurisdiction.
Ketorolac	0.5 mg/kg IV q6 h (max 15–30 mg/dose, max 5 days)	4–6 h	Inpatient acute severe postoperative pain; short-term use only due to renal/GI toxicity risk.	Hospital/short-term use; local pediatric authorization varies.
Diclofenac	0.5–1 mg/kg q8–12 h (max 150 mg/day)	1–2 h	Second-line acute inflammatory pain; limited pediatric evidence base.	Selected pediatric use; authorization varies by country.
Meloxicam	0.125–0.25 mg/kg once daily (indication/local labeling dependent)	~15–20 h	Chronic inflammatory pain/JIA; not a routine acute analgesic	Age limits and formulations vary by jurisdiction; verify local labeling.

**Table 3:** Pediatric opioids: dosing, age/regulatory limitations, and safety monitoring.

Drug	Mechanism	Typical Dose Range	Age Supported/Regulatory Status	Main Clinical Setting	Required Monitoring	Rescue/Safety Considerations
Morphine	μ-opioid agonist	Neonates: - slow infusion intravenously (bolus): 40–100 mcg/kg in at least 5–10 min, every 4–6 h; - infusion: 25–50 μg/kg (loading dose) followed by 5 μg/kg/h (preterm neonate); 50–100 μg/kg loading dose, followed by 10–20 μg/kg/h in case of neonates born at term. Children until 12 years: - bolus: 100–200 μg/kg maximum 6 times/daily; - infusion: after a bolus as loading dose, 10–30 μg/kg/h. 12–18 years: - bolus 2,5–10 mg maximum 6 times/daily; - infusion: after a bolus as loading dose, 10–30 μg/kg/h.	All ages (caution neonates)	Acute moderate-to-severe pain; palliative care	Respiratory rate, oxygenation, level of consciousness/sedation; hemodynamic monitoring as clinically indicated.	Use lowest effective dose; ensure protocol for opioid-induced respiratory depression and ready access to naloxone where parenteral/strong opioids are administered.
Oxycodone	μ-agonist	5 mg orally starting dose	≥12 years in Europe	Selected acute/palliative settings	Respiratory rate, oxygenation, level of consciousness/sedation; hemodynamic monitoring as clinically indicated.	Use lowest effective dose; ensure protocol for opioid-induced respiratory depression and ready access to naloxone where parenteral/strong opioids are administered.
Fentanyl	μ-agonist, high potency	Transdermal patch: Morphine 30–44 mg/daily = 12 mcg/h Morphine 45–134 mg daily = 25 mcg/h Higher morphine dose = a similar equianalgesic posology of adults Intravenous fentanyl 2–11 years: Starting dose 1–3 mcg/kg; Supplemental dose: 1–1.25 mcg/kg 12–17 years: like adults	≥2 years in Europe	Acute severe pain; selected palliative/transmucosal or transdermal use	Respiratory rate, oxygenation, level of consciousness/sedation; hemodynamic monitoring as clinically indicated.	Use lowest effective dose; ensure protocol for opioid-induced respiratory depression and ready access to naloxone where parenteral/strong opioids are administered.
Hydromorphone	μ-agonist	Oral: 1.3 or 2.6 mg every 4 h IV: not available in Italy. No data on pediatric patients in US.	≥12 years in Europe	Selected acute/palliative settings	Respiratory rate, oxygenation, level of consciousness/sedation; hemodynamic monitoring as clinically indicated.	Use lowest effective dose; ensure protocol for opioid-induced respiratory depression and ready access to naloxone where parenteral/strong opioids are administered.
CYP450, cytochrome P450.				Not recommended as routine pediatric analgesic	Respiratory rate, oxygenation, level of consciousness/sedation; hemodynamic monitoring as clinically indicated.	Use lowest effective dose; ensure protocol for opioid-induced respiratory depression and ready access to naloxone where parenteral/strong opioids are administered.
Codeine	μ-opioid agonism via active morphine metabolite	Not recommended for routine pediatric analgesia	Contraindicated <12 years; contraindicated <18 years after tonsillectomy/adenoidectomy under US restrictions	Avoid routine pediatric use; follow regional labeling	Respiratory status, sedation, CNS adverse effects if exposure occurs	CYP2D6 variability; risk of respiratory depression; tramadol also carries seizure/serotonergic risk.
Tramadol	Weak μ-opioid agonism + monoamine reuptake inhibition	Not recommended for routine pediatric analgesia	Contraindicated <12 years; contraindicated <18 years after tonsillectomy/adenoidectomy under US restrictions	Avoid routine pediatric use; follow regional labeling	Respiratory status, sedation, CNS adverse effects if exposure occurs	CYP2D6 variability; risk of respiratory depression; tramadol also carries seizure/serotonergic risk.

**Table 4:** Comparative overview of key gabapentinoids in pediatrics.

Drug	Mechanism of Action	Pharmacokinetic Profile	Excretion & Dosing Considerations
Gabapentin	Binds to α2δ subunit of presynaptic voltage-gated calcium channels; reduces excitatory neurotransmitter release.	Non-linear, saturable gastrointestinal absorption (L-amino acid transporter); variable bioavailability.	100% renal elimination; requires dose titration and thrice-daily dosing (q8 h); dose adjustment in renal impairment.
Pregabalin	High-affinity binding to α2δ subunit of presynaptic voltage-gated calcium channels.	Linear, dose-proportional absorption; high bioavailability (>90%); rapid peak plasma concentrations.	100% renal elimination; twice-daily dosing (q12 h); predictable PK profile compared to gabapentin.

**Table 5:** Practical mechanism- and setting-based framework for pediatric pain pharmacotherapy.

Pain Setting	Typical Age Group	Preferred Pharmacological Approach	Alternative/Adjunct	Main Indication	Evidence/Key Safety Issue
Acute nociceptive	Infants, children, adolescents	Acetaminophen or ibuprofen when appropriate	Selected NSAID	Trauma, dental, postoperative/inflammatory pain	Moderate–high; consider hepatic, renal, GI and hydration status
Severe acute pain	Age/setting dependent	Morphine or another protocol-based strong opioid	Fentanyl/selected opioid	Major trauma, postoperative or procedural pain	Moderate; respiratory depression requires structured monitoring
Chronic inflammatory nociceptive	Children/adolescents	NSAID according to indication and age	Disease-specific anti-inflammatory therapy	JIA and selected inflammatory conditions	Moderate; long-term GI/renal risk and disease-specific management
Neuropathic	Mainly older children/adolescents	Selected gabapentinoid or co-analgesic	TCA/SNRI/topical therapy in selected cases	Neuropathic syndromes/CIPN/nerve injury	Low; frequent off-label use, sedation/CNS effects
Nociplastic/chronic primary pain	Mainly adolescents	Non-pharmacological and multidisciplinary care first	Selected symptom-targeted pharmacotherapy	Fibromyalgia/functional pain	Low-very low; functional outcomes prioritized over pain elimination

## Data Availability

No new data were created or analyzed in this study. Data sharing is not applicable to this article.
